# Antioxidant Activities and Chemical Constituents of Flavonoids from the Flower of *Paeonia ostii*

**DOI:** 10.3390/molecules22010005

**Published:** 2016-12-23

**Authors:** Huifang Zhang, Xiaofang Li, Ke Wu, Mengke Wang, Pu Liu, Xinsheng Wang, Ruixue Deng

**Affiliations:** Chemical Engineering & Pharmaceutical College, Henan University of Science and Technology, Luoyang 471023, Henan, China; huifangz@163.com (H.Z.); xiaofl@163.com (X.L.); kewu@126.com (K.W.); mengkw@163.com (M.W.); xswang@126.com (X.W.)

**Keywords:** flavonoids of peony flower, *Paeonia ostii*, natural antioxidant, chemical constituents

## Abstract

*Paeonia ostii* is a traditional medicinal plant popularly used in China. This study intended to evaluate the antioxidant properties and the chemical components of the flavonoid-rich extracts from the flowers of *P. ostii*. The results showed that the flavonoid-rich extracts from the flowers of *P. ostii* had strong scavenging capacities on 2,2′-Azinobis-(3-ethylbenzthiazoline-6-sulphonate) (ABTS), hydroxyls, superoxide anions, and 1,1-diphenyl-2-picrylhydrazyl (DPPH) radicals in a dose-dependent manner. Five flavonoids, dihydrokaempferol (**1**), apigenin-7-*O*-β-d-glucoside (**2**), apigenin-7-*O*-β-d-neohesperidoside (**3**), kaempferol-7-*O*-β-d-glucopyranoside (**4**), and kaempferol-3-*O*-β-d-glucopyranosyl-7-*O*-β-d-glucopyranoside (**5**), were isolated from the flavonoid-rich extracts of the flowers of *P. ostii*. High-performance liquid chromatography (HPLC) analysis revealed that compounds **3** and **4** were abundant in the *P. ostii* flower and in flavonoid-rich extracts. The main components of the flower of *P. ostii* are flavonoids. The high antioxidant activity of the flavonoid-rich extracts may be attributed to the high content of flavonoids. The five isolated flavonoids were the primary antioxidant ingredients, and may play important roles in the strong antioxidant activities of this flower. Based on the obtained results, the flower of *P. ostii* could be a potential source of natural antioxidants in food and pharmaceutical applications.

## 1. Introduction

Flavonoids, which are widely distributed in plants, are naturally occurring phenolic compounds that possess many biological activities [1]. Flavonoids are also one of the most important bioactive ingredients of vegetables and fruits, and the intake of flavonoids can range between 50 and 800 mg·d^−1^ in a standard human diet [2,3,4]. Biological activities, such as the antiallergenic, antiviral, and anti-inflammatory activities of flavonoids, may originate from their antioxidant properties [1]. Thus, flavonoid antioxidants may perhaps have a great contribution to the prevention and treatment of many diseases.

Free radicals can damage the body by starting a chain reaction in important cellular compounds, such as DNA or cell membranes. Thus, free radicals are implicated in numerous pathological conditions, such as inflammation, metabolic disorders, cellular aging, reperfusion damage, and carcinogenesis [5,6,7]. Additionally, antioxidants can form a defense system in order to prevent free radicals from damaging the body. Many flavonoids are excellent free radical scavengers due to their strong abilities as hydrogen or electron donors [8,9]. Therefore, most studies have been carried out in order to find new sources of natural antioxidants from flavonoids because of their activities to scavenge free radicals [10,11].

The tree peony, commonly called the “King of Flowers” in China, belongs to the Paeoniaceae family, the *Paeonia* genus, and is a very popular, traditional Chinese ornamental plant due to its magnificent and colorful flowers [12]. The tree peony has a history of more than 1500 years of cultivation, which has led to various flower colorations and its wide distribution in China [13].

*Paeonia ostii* (Paeoniaceae), one of the several wild species of Chinese tree peony, is a woody shrub that is widely distributed in China [12]. The flower of *Paeonia ostii* was authorized as a new food resource by the National Health and Family Planning Commission of the People’s Republic of China in November 2013. Investigations into the chemical constituents of the pigment of the flowers have resulted in the identification of flavonoids [14]. Flavonoids are widely distributed in the *Paeonia* genus, and more than 40 flavonoids have been isolated from *Paeonia*. These compounds were mainly isolated from the flowers, petals, leaves, pollen, roots, and stamen of the plants [15,16]. Some flavonols were isolated from the flowers of *P. suffruticosa*, *P. lactiflora*, *P. potaninii*, *P. delavayi*, and *P. rochii* [17,18,19], anthocyanins were obtained from the flowers of *P. decra*, *P. lactiflora*, *P. tenuifolia*, *P. obovata*, *P. japonica*, *P. poeaninii*, *P. rockii*, and *P. decora* [18,19,20,21,22], and chalcones from the flowers of *P. suffruticosa*, *P. lutea*, *P. delavayi*, and *P. trollioides* [23,24]. Determination and analysis studies of nutritional ingredients showed that the seeds of *P. ostii* had abundant unsaturated fatty acids and monoterpene glycosides [25,26]. Moreover, studies that reported on the seeds of *P. rockii* resulted in the isolation of polyphenols with high activities in scavenging free radicals [27]. As they are a promising source of natural products, the petals of tree peony are used in aromatherapy and are also used as source of food and drink, such as in peony scented tea, peony flower drinks, peony flower sauce, and so on [28]. In addition, in traditional Chinese medicine, the tree peony flower is used in the treatment of gynecological diseases.

In the course of our studies on the bioactive constituents of tree peony flowers, we found that the ethanolic extracts of the flowers of *P. ostii* showed strong radical scavenging activities. In addition, further chemical constituents research showed that the flavonoids play important roles in radical scavenging activities. In this paper, we describe the enrichment of the total flavonoids, the radical scavenging activities of the flavonoid-rich extracts, the isolation and structure elucidation of the flavonoids isolated from the flavonoid-rich extracts, as well as the inhibitory effects of the isolated flavonoids on ABTS (2,2′-Azinobis-(3-ethylbenzthiazoline-6-sulphonate)), O•^2−^, DPPH (1,1-diphenyl-2-picrylhydrazyl), and •OH radicals. Additionally, the HPLC (High Performance Liquid Chromatography) content of the isolated flavonoids in dried flower and in flavonoid-rich extracts were also investigated.

The aim of the current investigation was to evaluate the flavonoid composition, antioxidant activities, and the content determination of the main isolated flavonoids of the flower of *P. ostii*. Hopefully, this study will provide sufficient experimental evidence for good antioxidant activity and the potential for further development and utilization of flavonoids from the flower of *P. ostii*.

## 2. Results and Discussion

### 2.1. Sample Extraction and Preparation

Total flavonoids of the flowers of *P. ostii* were extracted and enriched in this study. The fresh flower of *P. ostii* was powered and extracted with ethanol at room temperature for 48 h. The weight of the crude extract of this flower was 93.5 g. The extract (90 g) was suspended in 4 L of distilled water, and was then loaded onto a polyamide resin column for the enrichment of total flavonoids. Polyamide resin is a polyamide powder with a large specific area and a considerable particle size processed from polyamide chip [29]. Based on the affinity of the hydrogen bonding interactions [30], polyamide resin has attracted much attention due to its high adsorption and desorption capacities, and can be used to enrich flavonoids [29,31].

The total flavonoids of different ethanol concentration elution fractions were examined and shown in Table 1. The total flavonoid content was expressed as mg rutin equivalents per gram dry weight of each extract (mg·RE·g^−1^ DW), and the content of all samples tested in this study ranged from 43.4 to 276.0 mg rutin equivalent (RE)·g^−1^ dry weight. The result indicated that 50% and 60% ethanol elution have higher flavonoid contents than any other fractions. Through the enrichment of polyamide resin, the content of flavonoids of the fraction markedly increased [29,31]. Thus, the antioxidant activities and the chemical composition of these two fractions were studied further.

### 2.2. Antioxidant Activity

#### 2.2.1. ABTS Radical Scavenging Activity

**Figure 1:**
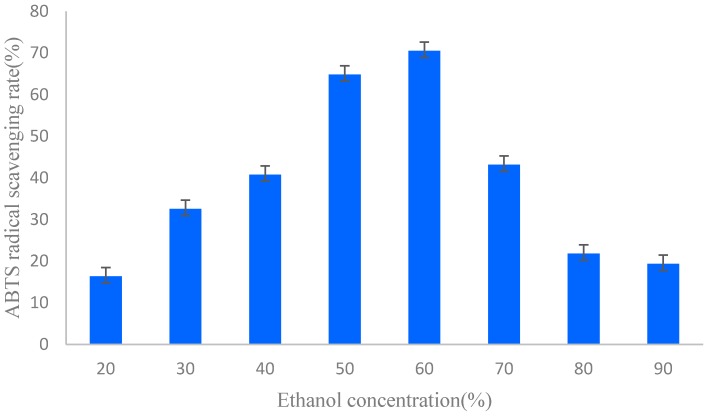
ABTS free radical scavenging rates of different ethanol concentration elution fractions.

The ABTS free radical scavenging activity of different ethanol concentration elution fractions were examined and are shown in Figure 1. Total antioxidant activity of different ethanol concentration elution fractions of the flower of *P. ostii* were assessed by measuring the reduction of the ABTS radical as a percentage of the inhibition at 734 nm. The extracts of different ethanol concentration elution fractions of the flower showed different inhibitory effects on ABTS free radicals. In addition, the scavenging effect of different ethanol concentration elution fractions on ABTS decreased in the order of: 60% ethanol elution fraction > 50% ethanol elution fraction > 70% ethanol elution fraction > 40% ethanol elution fraction > 30% ethanol elution fraction > 80% ethanol elution fraction > 90% ethanol elution fraction > 20% ethanol elution fraction, which were 70.47%, 64.82%, 43.15%, 40.78%, 32.54%, 21.78%, 19.32%, and 16.35%, respectively, at a 40 μg·mL^−1^ concentration. On the other hand, the results of the above-mentioned experiments indicated that, among the eight fractions, 50% and 60% ethanol elutions contained the highest flavonoid contents of all fractions. High correlations between the content of flavonoids in fractions (Table 1) and the results of antioxidant tests (Figure 1) might be indicative of the possible, high antiradical activity of the flavonoids. Flavonoids, perhaps, contribute greatly to the antioxidant activity of the flower of *P. ostii*. ABTS is generally used for testing the preliminary radical scavenging activity of antioxidant compounds or plant extracts, and can truly reflect the antioxidant contents in a variety of foods [32]. Flavonoids are widely distributed in genus *Paeonia*, and the methanolic extracts from the roots of *P. suffruticosa* contain the most flavonoids, which may explain its high free radical scavenging activity [33]. Moreover, the antioxidant activities of flavonoid content may partly be associated with their abilities to scavenge free radicals, and the higher the flavonoid content, and the stronger the radical scavenging activity [33].

The 50% and 60% fractions were combined into one fraction (17.86 g), and were considered to be the flavonoid-rich extract of *P. ostii*, which was then used for the further antioxidant assays and chemical composition isolation and analysis. 

**Figure 2:**
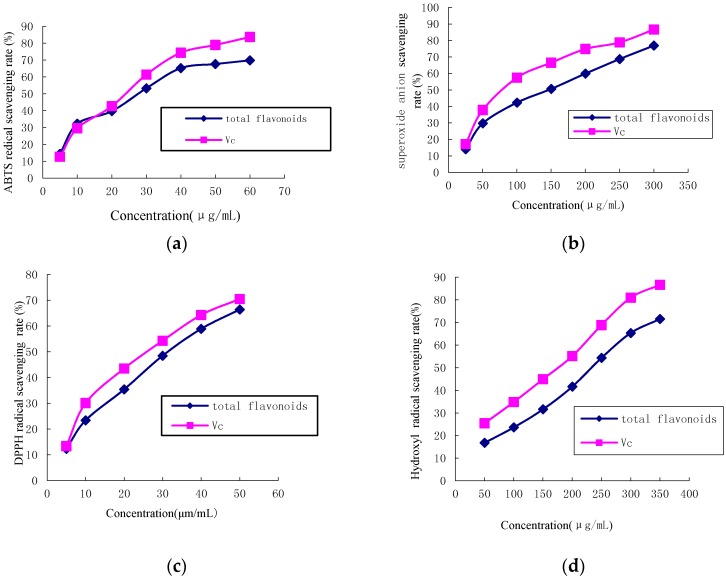
Effects of total flavonoids on radical scavenging rates: (**a**) Curve of ABTS scavenging rate; (**b**) curve of superoxide anion scavenging rate; (**c**) curve of DPPH radical scavenging rate; (**d**) curve of •OH scavenging rate.

The antioxidant activity of flavonoid-rich extract from the flower of *P. ostii* was assessed by measuring the reduction of the ABTS radical as a percentage of the inhibition at 734 nm. The ABTS scavenging activities of various concentrations of total flavonoids, from 5 to 60 μg·mL^−1^, on ABTS radicals were shown in Figure 2a. The inhibition was found to be concentration dependent, as the scavenging capability increased with sample concentration. Vitamin C (Vc) exhibited a high radical scavenging rate when the concentrations were over 20 μg·mL^−1^.

#### 2.2.2. Superoxide Anion Free Radical Scavenging Activity

Figure 2b shows the inhibitory effect of the flavonoid-rich extracts from the flower of *P. ostii* on superoxide radical generation. Experiments were performed under controlled conditions. The results indicated that, in the range of 50–350 μg·mL^−1^, the flavonoid-rich extracts from the flower of *P. ostii* interfered with superoxide anion free radicals in a dose-dependent manner. The IC_50_ values of flavonoid-rich extracts and Vc were 131.12 ± 4.56 μg·mL^−1^ and 72.34 ± 2.56 μg·mL^−1^, respectively. More than 1.8 times the concentration of flavonoid-rich extracts was needed to have an effect on superoxide anion free radicals relative to Vc in this study. There were reports on the superoxide anion free radicals of the flower of *P. ostii*. The IC_50_ value of methanol extract from the flowers of *P. suffruticosa* on the superoxide anion free radical was 199.0 μg·mL^−1^ [34], which was higher than that of the purified flavonoid-rich extract obtained in this study. 

#### 2.2.3. DPPH Radical Scavenging Activity

Various methods have been designed to measure antioxidant power. The reaction of the colored DPPH radical is a popular method to evaluate the strength of antioxidants. Figure 2c shows the radical scavenging ability of flavonoid-rich extracts on DPPH. At concentrations from 5.0 to 50.0 μg·mL^−1^, the DPPH radical scavenging ability of flavonoid-rich extracts ranged from 13.4% to 68.6%, while that of Vc was 15.7%–72.8%. IC_50_ values were 32.6 ± 0.54 μg·mL^−1^ and 26.4 ± 0.61 μg·mL^−1^ for flavonoid-rich extracts and Vc, respectively. The flavonoid-rich extracts had a strong scavenging activity on DPPH radicals, based on the fact that the effective concentration to inhibit radical scavenging ability was slightly higher than that of pure Vc. The free radical scavenging activity of extracts from the flower of genus *Paeonia* has been demonstrated in several studies. The free radical scavenging activity of extracts from the flower of genus *Paeonia* has been demonstrated in several studies. The antioxidant activity of the extracts from the flowers of the Zhongyuan tree penoy cultivars varied from 7.66 to 31.36 mg Vc equivalents·g^−1^ for the DPPH assay, and for *P. ostii* (Feng dan bai) was 8.63 mg Vc equivalents·g^−1^ for DPPH [33]. The scavenging activity of crude extract from the flower of *P. suffruticosa* on DPPH radical was 4.77% of maximum inhibition at 20 μg·mL^−1^ concentration [35]. Therefore, the purified flavonoid-rich extract obtained in this study was superior to most of the other reported values.

The DPPH radical assay is based on the reduction of alcoholic DPPH solution in the presence of a hydrogen donating antioxidant [36]. The 2,2-diphenyl-1-picrylhydrazyl radical (DPPH•) can be reduced to its hydrazine form (DPPH-H) by antioxidants because of their hydrogen-donating abilities [37]. Like many other polyphenols, flavonoids are excellent free radical scavengers because they are highly reactive as hydrogen or electron donors [8,9]. Thus, commercially available DPPH• is becoming widely used to investigate the free radical scavenging activities of flavonoid-rich extracts and flavonoids.

#### 2.2.4. Hydroxyl Radical Scavenging Activity

As an extremely active free radical in biological systems, the hydroxyl radical can cause oxidative damage to biological macromolecules, including lipids, proteins, and nucleic acids [38]. Antioxidants can prevent damage to the hydroxyl radical of biological macromolecules. Additionally, the scavenging capacity on hydroxyl radicals is directly related to the antioxidant capability [39]. The radical scavenging results of flavonoid-rich extracts on hydroxyl radicals are shown in Figure 2d. The hydroxyl radical scavenging activity of flavonoid-rich extracts and Vc both increased with an increase in concentrations, and the scavenging activity at 200 μg·mL^−1^, for flavonoid-rich extracts and Vc, were 42.5% and 57.1%, respectively. In addition, Vc showed an excellent scavenging activity with an IC_50_ value of 162.12 ± 3.15 μg·mL^−1^, and flavonoid-rich extracts were also observed to have scavenging activity with an IC_50_ value of 233.54 ± 6.47 μg·mL^−1^, which was higher than that of methanol extract from the flowers of *P. suffruticosa* with the IC_50_ value 193.18 μg·mL^−1^ [35]. Flavonoid-rich extracts remarkably inhibited hydroxyl radicals. 

### 2.3. Structural Elucidation of the Isolated Compounds

**Figure 3:**
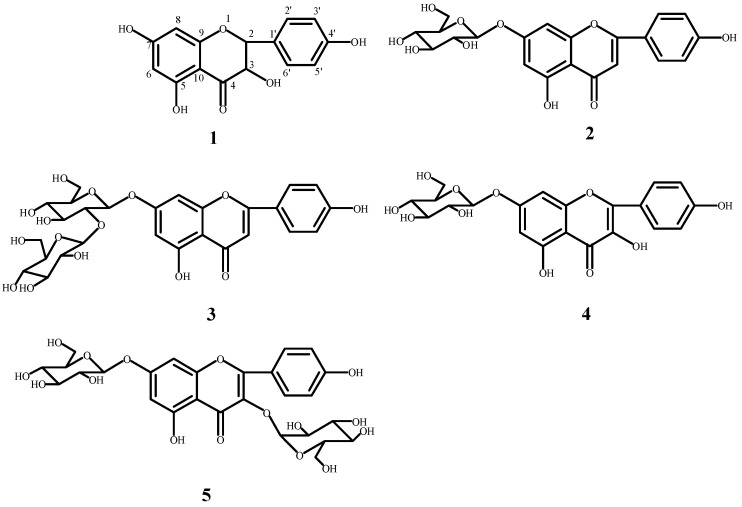
The structures of compounds: **1**–**5** (dihydrokaempferol (**1**); apigenin-7-*O*-β-d-glucoside (**2**); apigenin-7-*O*-β-d-neohesperidoside (**3**); kaempferol-7-*O*-β-d-glucopyranoside (**4**); kaempferol-3-*O*-β-d-glucopyranosyl-7-*O*-β-d-glucopyranoside (**5**)).

The chemical structures of the isolated compounds were identified according to their ESI-MS (Electrospray Ionization Mass Spectrometry), ^1^H-NMR (Proton Nuclear Magnetic Resonance) and ^13^C-NMR (Carbon Nuclear Magnetic Resonance) data. The chemical structures of the isolated compounds are shown in Figure 3. The data of each compound are given as follows:

*Dihydrokaempferol* (**1**), light yellow crystallized powder. Additionally, it has a strong ultraviolet absorption under UV254. ESI-MS *m*/*z*: 287 [M − H]^−^ (C_15_H_12_O_6_). ^1^H-NMR (400 MHz, CD_3_OD) δ: 4.54 (1H, d, *J* = 11.6 Hz, H-3), 4.97 (1H, d, *J* = 11.6 Hz, H-2), 5.87 (1H, d, *J* = 1.59 Hz, H-6), 5.91 (1H, d, *J* = 1.59 Hz, H-8), 7.35 (2H, d, *J* = 8.56 Hz, H-2′, 6′), 6.82 (2H, d, *J* = 8.56 Hz, H-3′, 5′). ^13^C-NMR (100 Hz, CD_3_OD) δ: 84.99 (C-2), 73.65 (C-3), 198.53 (C-4), 165.34 (C-5), 97.32 (C-6), 168.76 (C-7), 96.28 (C-8), 164.57 (C-9), 101.85 (C-10), 129.30 (C-1′), 130.38 (C-2′), 116.14 (C-3′), 159.25 (C-4′), 116.14 (C-5′), 130.38 (C-6′). Based on the above-mentioned data and a comparison with data from the literature [40], compound **1** was identified as dihydrokaempferol.

*Apigenin-7-O-β-d-glucoside* (**2**) was obtained as light yellow crystallized powder, and showed strong ultraviolet absorption under UV254. ESI-MS *m*/*z*: 434 [M − H]^−^ (C_21_H_21_O_10_). ^1^H-NMR (400 MHz, DMSO) δ: 6.88 (1H, s, H-3), 6.45 (1H, d, *J* = 1.59 Hz, H-6), 6.83 (1H, d, *J* = 1.59 Hz, H-8), 7.96 (2H, d, *J* = 8.8 Hz, H-2′, 6′), 6.97 (2H, d, *J* = 8.8 Hz, H-3′, 5′). ^13^C-NMR (100 MHz, CD_3_OD) δ: 164.23 (C-2), 103.07 (C-3), 181.97 (C-4), 156.91 (C-5), 94.81 (C-6), 162.92 (C-7), 99.48 (C-8), 161.35 (C-9), 105.30 (C-10), 120.98 (C-1′), 128.59 (C-2′), 115.97 (C-3′), 161.08 (C-4′), 115.97 (C-5′), 128.59 (C-6′), 99.86 (C-1′′), 73.07 (C-2′′), 76.40 (C-3′′), 69.52 (C-4′′), 77.14 (C-5′′). Based on the data, and a comparison with data from the literature [41], compound **2** was identified as apigenin-7-*O*-β-d-glucoside.

*Apigenin-7-O-β-d-neohesperidoside* (**3**) was obtained as an amorphous yellow powder, and exhibited strong ultraviolet absorption under UV254. ESI-MS *m*/*z*: 577 [M − H]^−^ (C_27_H_30_O_14_). ^1^H-NMR (400 MHz, CD_3_OD) δ: 6.60 (1H, s, H-3), 6.40 (1H, d, *J* = 1.8 Hz, H-6), 6.71 (1H, d, *J* = 1.8 Hz, H-8), 7.82 (2H, d, *J* = 8.2 Hz, H-2′, 6′), 6.89 (2H, d, *J* = 8.2 Hz, H-3′, 5′). ^13^C-NMR (100 MHz, CD_3_OD) δ: 166.67 (C-2), 104.12 (C-3), 183.97 (C-4), 162.93 (C-5), 100.97 (C-6), 164.34 (C-7), 95.90 (C-8), 158.90 (C-9), 107.01 (C-10), 122.96 (C-1′), 129.67 (C-2′), 117.04 (C-3′), 162.88 (C-4′), 117.04 (C-5′), 129.63 (C-6′), 99.75 (C-1′′), 79.03 (C-2′′), 79.03 (C-3′′), 71.40 (C-4′′), 78.30 (C-5′′), 62.45 (C-6′′), 102.54 (C-1′′′), 72.22 (C-2′′′), 72.22 (C-3′′′), 73.99 (C-4′′′), 70.01 (C-5′′′), 18.29 (C-6′′′). Based on the data, and a comparison with data from the literature [42], compound **3** was identified as apigenin-7-*O*-β-d-neohesperidoside.

*Kaempferol-7-O-β-d-glucopyranoside* (**4**) was obtained as an amorphous yellowish powder, and presented strong ultraviolet absorption under UV254. ESI-MS *m/z*: 447 [M − H]^−^ (C_21_H_20_O_11_). ^1^H-NMR (400 MHz, DMSO) δ: 6.79 (1H, d, *J* =2.2 Hz, H-8), 6.44 (1H, d, *J* = 2.2 Hz, H-6), 8.06 (2H, d, *J* = 8.9 Hz, H-2′, 6′), 6.89 (2H, d, *J* = 8.9 Hz, H-3′, 5′). ^13^C-NMR (100 Hz, DMSO) δ: 147.3 (C-2), 135.9 (C-3), 175.9 (C-4), 1620.1 (C-5), 98.7 (C-6), 162.5 (C-7), 94.3 (C-8), 155.6 (C-9), 104.6 (C-10), 121.4 (C-1′), 129.5 (C-2′, 6′), 78.0 (C-3′, 5′), 159.2 (C-4′). Based on the data, and a comparison with data from the literature [43], compound **4** was identified as kaempferol-7-*O*-β-d-glucopyranoside.

*Kaempferol-3-O-β-d-glucopyranosyl-7-O-β-d-glucopyranoside* (**5**) was obtained as an amorphous yellowish powder, and has strong ultraviolet absorption under UV254. ESI-MS *m*/*z*: 609 [M − H]^−^ (C_27_H_30_O_16_). ^1^H-NMR (400 MHz, CD_3_OD) δ: 6.445 (1H, d, *J* = 2.1 Hz, H-6), 6.791(1H, d, *J* = 2.1 Hz, H-8), 8.07 (2H, d, *J* = 8.9 Hz, H-2′, 6′), 6.90 (2H, d, *J* = 8.9 Hz, H-3′, 5′). ^13^C-NMR (100 MHz, CD_3_OD) δ: 155.97 (C-2), 133.41 (C-3), 177.60 (C-4), 160.81 (C-5), 99.29 (C-6), 162.77 (C-7), 94.6 (C-8), 155.97 (C-9), 105.60 (C-10), 120.73 (C-1′), 130.60 (C-2′), 115.11 (C-3′), 160.09 (C-4′), 115.11 (C-5′), 130.60 (C-6′), 99.64 (C-1′′), 73.03 (C-2′′), 76.37 (C-3′′), 69.51 (C-4′′), 77.12 (C-5′′), 60.76 (C-6′′), 100.63 (C-1′′′), 74.17 (C-2′′′), 76.63 (C-3′′′), 69.85 (C-4′′′), 77.52 (C-5′′′), 60.57 (C-6′′′). Based on the data, and a comparison with data from the literature [44], compound **5** was identified as kaempferol-3-*O*-β-d-glucopyranosyl-7-*O*-β-d-glucopyranoside.

Flavonoids are widely distributed in the flowers, petals, leaves, pollen, roots, and stamen of the plants of genus *Paeonia* [15,16]. Compounds **2**–**5** were previously isolated from the flowers of genus *Paeonia,* kaempferol-3-*O*-β-d-glucopyranosyl-7-*O*-β-d-glucopyranoside was isolated from the petals of *P. lactiflora* and *P. suffruticosa* [15,16,20], and apigenin-7-*O*-β-d-neohesperidoside, kaempferol-7-*O*-β-d-glucopyranoside, and apigenin-7-*O*-β-d-glucoside were obtained from the flower of *P. suffruticosa* [21,22].

### 2.4. Antioxidant Capacities of Isolated Compounds

The activities of these five isolated compounds are shown in Table 2. The five isolated compounds showed significant radical scavenging activities against ABTS, O•^2−^, DPPH and •OH radicals. Amongst these, compounds **1** and **5** possessed the strongest radical scavenging activities against all tested radicals, including ABTS, O•^2−^, DPPH and •OH radicals, with EC_50_ values of 25.4, 75.8, 24.6, and 141.9 μg·mL^−1^ for **1** and 23.5, 69.4, 20.9, and 121.8 for **5**, respectively. Based on the EC_50_ values against ABTS and •OH radicals, the activity order of the five isolated flavonoids was as follows: Compound **5** > compound **1** > compound **4** > compound **2** > compound **3**. The activity of compound **5** surpassed that of the positive control drug (Vc).

### 2.5. HPLC Analysis

The flavonoid-rich extracts and the isolated flavonoids showed significant radical scavenging activity. In order to further understand the relationship between the content of isolated flavonoids in the flavonoid-rich extracts and the radical scavenging activities of the total flavonoids, the content of the isolated flavonoids in the dried flower and flavonoid-rich extracts of *P. ostii* were analyzed. Five isolated flavonoids, dihydrokaempferol (**1**), apigenin-7-*O*-β-d-glucoside (**2**), apigenin-7-*O*-β-d-neohesperidoside (**3**), kaempferol-7-*O*-β-d-glucopyranoside (**4**), and kaempferol-3-*O*-β-d-glucopyranosyl-7-*O*-β-d-glucopyranoside (**5**), were quantified.

**Figure 4:**
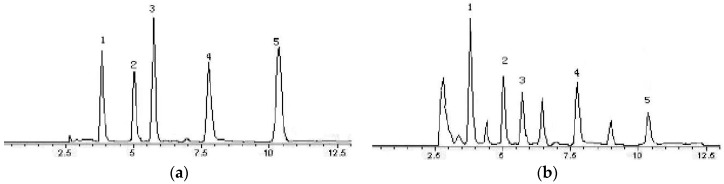
Chromatograms of chemical reference substances (**a**) and sample (**b**) in 360 nm (1—Apigenin-7-*O*-β-d-neohesperidoside (**3**); 2—kaempferol-3-*O*-β-d-glucopyranosyl-7-*O*-β-d-glucopyranoside (**4**); 3—kaempferol-7-*O*-β-d-glucopyranoside (**5**) 4—apigenin-7-*O*-β-d-glucopyranoside (**2**); 5—dihydrokaempferol (**1**)).

The representative chromatograms of the standard mixture solution and flavonoid-rich extracts are depicted in Figure 4. The HPLC method provided repeatable and good separations for the five isolated flavonoid compounds. Based on the chromatograms, dihydrokaempferol (**1**), apigenin-7-*O*-β-d-glucoside (**2**), apigenin-7-*O*-β-d-neohesperidoside(**3**), kaempferol-7-*O*-β-d-glucopyranoside (**4**), and kaempferol-3-*O*-β-d-glucopyranosyl-7-*O*-β-d-glucopyranoside (**5**) were well separated at a retention time of 3.78 min, 5.0 min, 5.69 min, 7.74 min, and 10.42 min, respectively. In addition, the contents of individual flavonoids identified in the *P. ostii* flower, and in flavonoid-rich extracts, are presented in Table 3. According to the HPLC results, compounds **3** and **4** were abundant in *P. ostii* flower and in flavonoid-rich extracts (the contents of compounds **3** and **4** were 10.25 ± 0.35 and 6.44 ± 0.45 mg·g^−1^ of dry sample, respectively). Compounds **1**–**5** perhaps contribute greatly to the strong antioxidant activity of this flower. The high antioxidant activity of the extracts may be attributed to their high content of flavonoids.

## 3. Materials and Methods

### 3.1. Plant Material

Fresh flowers of *P. ostii*, originating in Henan, China, were collected from Luoyang Tuqiao flower and seeding Co. Ltd., Luoyang, Henan, China. They were identified by teacher Zhipeng Wei (CEO of Luoyang Tuqiao flower and seeding Co., Ltd., Luoyang, Henan, China) in May 2014. A voucher specimen was deposited in the Specimens Hall of Natural Resources of Funiu Mountains, Henan University of Science and Technology.

### 3.2. Reagent and Chemicals

All chemicals used were of analytical or HPLC grade. Lecithinum, 1,1-diphenyl-2-picrylhydrazyl radical (DPPH), [2,20-azinobis-(3-ethylbenzothiazoline-6-sulfonic acid)] diammonium salt (ABTS), nicotinamide adenine dinucleotide (NADH), phenazine methyl sulfate (PMS), nitrotetrazolium blue chloride (NBT), and Vitamin C were obtained from Aladdin Industrial Corporation (Shanghai, China). 2-Hydroxybenzoic acid, disodium hydrogen phosphate dodecahydrate, sodium dihydrogen phosphate, ferrous sulfate, and hydrogen peroxide were purchased from Tianjin Kermel Chemical Reagent Co., Ltd. (Tianjin, China). Toypear HW-40C (Tosoh, Japan) was supplied by Beijng Greenberbs Science and Technology Development Co., Ltd. (Beijing, China). Silica gel GF254 plates and silica gel were purchased from Qingdao Marine Chemical Co. (Qingdao, China). The water was purified using a ULUP-111-10T water purification system (Chengdu, China).

### 3.3. Apparatus

All of the ^1^H and ^13^C nuclear magnetic resonance (NMR) spectra were recorded on a Bruker Avance 400 spectrometer (Karlsruhe, Germany), using tetramethylsilane (TMS) as an internal standard, at 400 (^1^H) and 100 MHz (^13^C). The chemical shifts in the NMR spectra were recorded as δ values. ESI-MS was obtained on a Waters Alliance 2695, Quattro Micro TM ESI instrument (Milford, MA, USA). 

Semi-preparative HPLC was performed using a Waters 600 with Waters TP pump, UV-2487 detector (Milford, MA, USA), and a YMC-Pack ODS-A column (SH-343-5, Tokyo, Japan). Column chromatography (CC) was performed on silica gel (Qingdao Marine Chemical Co., Ltd., Qingdao, China) or Toyopearl HW-40 (Tosoh, Tokyo, Japan). Thin-layer chromatography (TLC) was performed on silica gel GF254 plates (Qingdao Marine Chemical Co., Ltd., Qingdao, China), visualization was done under UV light and by spraying with Ce_2_SO_4_ or phosphomolybdic acid hydrate, followed by heating. HPLC analysis was carried out on Agilent 1100 (Agilent, Palo Alto, CA, USA) equipped with a photo-diode-detector (DAD), quarternary Pump G1311A, an autosampler G1313A, a G1322A vacuum degasser, and the Agilent HPLC workstation. The UV absorbance was measured at 510 nm using an ultraviolet-visible spectrophotometer, UV-2600 (Shimadzu, Suzhou, China).

### 3.4. Sample Extraction and Preparation

The procedure of extraction of total flavonoids, as described in the literature, has been modified [45]. The fresh flower of *P. ostii* was collected, and the peduncle, receptacles, and calyx were removed from the large flower, leaving only the petals of the flower. The 30 Kg petals were powered, and extracted thrice, each time with 20 L of ethanol at room temperature for 48 h. The combined ethanol extract was filtered and evaporated at 60 °C under reduced pressure using a vacuum rotary evaporator (Yuhua RE-2000A, Zhengzhou, China). The weight of the crude extract of this flower was 93.5 g.

The crude extract (90 g) was suspended in distilled water and subsequently filtered using filter papers. The filtrates were loaded onto the polyamide column (inner diameter 15 cm, length 120 cm, 8000 mL) for total flavonoid enrichment at a flow rate of 4 BV/h. After the polyamide resin was saturated with flavonoid compounds, the resin column was washed with distilled water and 20%, 30%, 40%, 50%, 60%, 70%, 80%, and 90% ethanol (*v*/*v*) with isocratic modes at a flow rate of 4.0 BV/h. The content of total flavonoids in each desorbed fraction was analyzed by UV-2600 (Ultraviolet-visible 2600) spectrophotometer. The desorbed fractions were concentrated in an evaporator at 60 °C under vacuum. The dried products of each fraction were weighed, and the content and recovery yields of total flavonoids were calculated.

### 3.5. Determination of Total Flavonoid Content

Using the aluminum chloride colorimetric method, the total flavonoid contents were determined, based on the literature reported, with some modifications [46]. Briefly, 4.0 mL 70% aqueous ethanol (*v*/*v*) and 0.3 mL NaNO_2_ (5%, *w*/*v*) were added to 1.0 mL of sample solution. After shaking, the mixture was incubated at room temperature for 6 min, and then 0.3 mL AlCl_3_ (10%, *w*/*v*) and 4.0 mL NaOH (1 mol/L) were added, followed by the addition of distilled water to reach a final volume of 10.0 mL. The solution was shaken and then allowed to stand at room temperature for 15 min. Finally, the absorbance was measured in 1 cm quartz cuvettes at 510 nm against the control with a UV-2600 spectrophotometer (Shimadzu, Suzhou, China). The control contained all the reaction reagents, except for the test sample. A calibration curve was measured, using a standard of rutin, the regression equation was: *y* = 0.02018*x* + 0.16 (*R*^2^ = 0.99925) (where *y* is the absorption and *x* is the rutin concentration in μg·mL^−1^). The results of total flavonoid contents of the extracts were calculated from the calibration curve and expressed as mg rutin equivalents per gram dry weight of extract (mg·RE·g^−1^).

### 3.6. Antioxidant Activity

#### 3.6.1. ABTS Radical Scavenging Activity

ABTS radical scavenging activity was determined using the method described in the literature with slight changes [41]. A stock solution of ABTS radical cations was prepared by mixing 50 mL 2.0 mmol·L^−1^ ABTS and 200 mL 70 mmol·L^−1^ potassium persulfate in an amber bottle. To produce ABTS radical cations, the mixture was incubated in the dark at room temperature and left to stand for 12–16 h, and then was diluted with phosphate buffer (pH = 7.4) and measured using a spectrophotometer absorbance at 735 nm of 0.7 ± 0.02. One hundred microliters of extract solution at different concentrations was added to 1.9 mL of ABTS reaction system. After being mixed and placed at room temperature in the dark for 6 min, the solution was measured for absorbance at 735 nm in a UV-2600 spectrophotometer. The sample (100 μL) was diluted with 95% ethanol (*v*/*v*) to give 20%–80% inhibition of blank absorbance. Vitamin C (Vc) was used as a positive control. The capability to scavenge the ABTS radicals was calculated based on the following equation:

(1) I%=[1−As/Ac]×100%

where the *Ac* is the absorbance of the ABTS solution without sample (100 μL distilled water + 1.9 mL ABTS) and *As* is the absorbance of the test sample mixed with ABTS solution (100 μL sample + 1.9 mL ABTS).

#### 3.6.2. Superoxide Anion Free Radical Scavenging Activity

The superoxide radical scavenging activity of the samples was assessed using the NADH-PMS-NBT (Nicotinamide Adenine Dinucleotide-Phenazine Methyl Sulfate-Nitrotetrazolium Blue Chloride) method described in the literature with some modifications [47]. Different concentrations of samples were prepared by dissolving samples in Tris-HCl buffer solution (pH 8.0, 0.05 mmol·L^−1^). The sample solutions were prepared by mixing with 0.5 mL nitroblue tetrazolium solution (NBT, 0.3 mmol·L^−1^), 0.5 mL phenazine methyl sulfate solution (0.06 mmol·L^−1^), and 0.5 mL of NADH solution (0.468 mmol·L^−1^), respectively. The reaction mixture was placed at room temperature in the dark for 5 min. The absorbance of the reaction mixtures was recorded at 560 nm with a UV-2600 spectrophotometer. The superoxide scavenging activity was expressed as percentage of inhibition. Results were calculated using the following formula:

(2) I%=[1−As/Ac]×100%

*Ac* was the absorbance of the blank without sample, while *As* was the absorbance of the sample.

#### 3.6.3. DPPH Radical Scavenging Activity Assay

The DPPH free radical scavenging activity of the flavonoid-rich extracts was determined according to a previously reported method with some modifications [48]. Two milliliters of the total flavonoid solutions, at different concentrations, were mixed with 2.0 mL of freshly-prepared DPPH solution (0.04 mg·mL^−1^ in 95% ethanol). After being thoroughly shaken and placed in the dark for 20 min, the mixture was transferred to a centrifuge tube and then centrifuged at 3500 rpm at 25 °C for 20 min. The supernatant was decanted, and the absorbance was measured at 517 nm against a blank using a UV-2600 spectrophotometer. Vitamin C was used as a reference compound in the same concentration range as the test compounds. A control solution was prepared in the same manner as the assay mixture without the tested compound. The capability of scavenging DPPH radicals as a percentage of DPPH remaining in the resulting solution was determined using the following equation:

(3) I%=[1−(Ai−Aj)/Ac]×100%

where the *Ac* is the absorbance of the DPPH solution without sample (2.0 mL 95% ethanol + 2.0 mL DPPH); *Ai* is the absorbance of the test sample mixed with DPPH solution (2.0 mL sample + 2.0 mL DPPH) and *Aj* is the absorbance of the test sample (2.0 mL sample + 2.0 mL 95% ethanol).

#### 3.6.4. Hydroxyl Radical Scavenging Activity

The Hydroxyl radical scavenging activity was performed using the method [49] with some minor changes. All solutions were freshly prepared. Two milliliters of various concentrations of sample were mixed with 2 mL of FeSO_4_ (6 mmol·L^−1^) and 2 mL of H_2_O_2_ (6 mmol·L^−1^). After being shaken, the mixture was placed at room temperature for 10 min, and followed by the addition of 2 mL salicylic acid (6 mmol·L^−1^). The mixture was shaken and incubated at room temperature in the dark for 30 min. The absorbance of the reaction mixture was measured at 510 nm using a UV-2600 spectrophotometer. Extra pure water was used as a control solution, and Vc was used as reference. The ability to scavenge hydroxyl radicals was calculated using the following equation:

(4) I%=[1−As/Ac]×100%

where the *Ac* is the absorbance of solution without sample and *As* is the absorbance of the test sample.

### 3.7. Isolation and Purification of Bioactive Compounds

The 50% ethanol and 60% ethanol fractions were further purified because of the higher content of total flavonoids and the strong antioxidant activities. Fifteen grams of 50% ethanol and 60% ethanol fractions of mixed powder was fractionated by normal-phase silica gel column chromatography and eluted with CHCl_3_/MeOH using a gradient solvent system (CHCl_3_/MeOH (9:1, 7:1, 5:1, 4:1, 1:2, *v*/*v*)) to obtain eight fractions (Fr. 1–8).

Fr. 3 (1800 mg) was subjected to column chromatography over silica gel using a stepwise gradient elution of petroleum ether/ethyl acetate (8:1–1:3, *v*/*v*) to get five subfractions (Subfr. 3.1–3.5). Subfr. 3.4 (300 mg) was separated by semi-preparative HPLC (ODS, MeOH/H_2_O (60:40, *v*/*v*)) to obtain compound **1** (28.0 mg).

Fr. 5 (1500 mg) was chromatographed using silica gel column chromatography to get five subfractions (Subfr. 5.1–5.5) using petroleum ether/acetone (1:3, *v*/*v*). Subfraction 5.4 (350 mg) was purified using a Toyperl HW-40C column with CHCl_3_/MeOH (2:1, *v*/*v*) eluent to give compound **2** (35.4 mg).

Compound **3** (22.3 mg) was obtained by HPLC (ODS-A; MeCN/H_2_O 3:7 (*v*/*v*), 3.0 mL·min^−1^) from Fr. 6 (800 mg).

Fr. 7 (980 mg) was separated using semi-preparative HPLC (ODS, MeOH/H_2_O (45:55, *v*/*v*)) to afford six subfractions (Subfr. 7.1–7.6). Subfraction 7.3 was then purified using HPLC (ODS-A, MeOH/H_2_O (4:6, *v*/*v*), 3.0 mL·min^−1^) to obtain compounds **4** (35.6 mg) and **5** (27.8 mg).

### 3.8. Identification of the Isolated Compounds

The chemical structures of the isolated compounds from the fractions with antioxidant activity were identified using Electrospray Ionization Mass Spectrometry (ESI-MS), ^1^H Nuclear Magnetic Resonance Spectroscopy (^1^H-NMR), and ^13^C Nuclear Magnetic Resonance Spectroscopy (^13^C-NMR) spectra with methanol or dimethyl sulfoxide (DMSO) used as solvent, and tetramethylsilane (TMS) used as internal standard. 

### 3.9. Flavonoids Compounds Analysis by HPLC

The dried and powered *P. ostii* flower was extracted twice with 75% ethanol (*v*/*v*) at room temperature. The resulting extract was prepared as a solution in methanol, and the flavonoid-rich extracts and the isolated flavonoid compounds were also dissolved in methanol. All the samples were filtered through a 0.45 μm filter, and then were injected into a Agilent 1100 HPLC system. The samples were eluted by using a Zorbax SB C18 column (4.6 × 250 mm, 5 µm) and detected at 360 nm. The column temperature was set at 30 °C. The gradient elution was set as follows: Eluent A was 0.5% acetic acid, and eluent B was acetonitrile. Gradient, 0.0 min (40% A and 60% B)—7 min, (50% A and 50% B)—15 min (60% A and 40% B). The flow rate was kept constant at 1 mL/min, and the injection volume was 10 μL for each run, which was performed in triplicate. The purity values of the isolated flavonoids were calculated using the area normalization method.

Flavonoids were identified by comparing their retention times with those of pure standards and by spiking the samples with standard solutions. Calibration curves were performed by injecting the standards three times at five different concentrations. Results were acquired, processed using the Agilent G2170BA software, and expressed as mg·g^−1^ sample of dry weight.

### 3.10. Data Analysis

All experiments were carried out in triplicate (*n* = 3). The results were analyzed and expressed as mean ± standard deviation (SD). Statistical analysis was done using one-way analysis of variance.

## 4. Conclusions

In the Henan, Shandong, Anhui, Sichuan, and Gansu provinces, China, the flower of *P. ostii* is usually used for eating and drinking, and also for aromatherapy in order to purify and harmonize the body and soul [12]. This present study focused on the antioxidant activity and the chemical composition of the flavonoid-rich extracts from the flower of *P. ostii*. The results showed that 50% and 60% ethanol elutions, which had high contents of total flavonoids, from the polyamide resin column of this plant, showed a high potential for antioxidant activities. Additionally, the antioxidant activities were well correlated with the contents of the flavonoids. An investigation on the chemical constituents was carried out. Five flavonoid compounds, dihydrokaempferol, apigenin-7-*O*-β-d-glucoside, apigenin-7-*O*-β-d-neohesperidoside, kaempferol-3-*O*-β-d-glucopyranosyl-7-*O*-β-d-glucopyranoside, and kaempferol-7-*O*-β-d-glucopyranoside, were isolated and identified from the flavonoid-rich extracts. All the isolated flavonoids possessed significant ABTS, O•^2−^, DPPH and •OH radical scavenging activities. HPLC analysis showed that the main flavonoid components in the flavonoid-rich extracts and in the flower of *P. ostii* are very high, which perhaps contribute greatly to the antioxidant activity of this flower. Flavonoids are implicated in the maintenance of health because of their antioxidant properties and other bioactivities [50,51]. Radical scavenging activities are very important due to the deleterious role of free radicals in foods and biological systems. Excessive formation of free radicals accelerates the oxidation of lipids in foods and decreases food quality and consumer acceptance [52]. Our results indicated that the extract from the flower of *P. ostii* could be a natural antioxidant source, and might have potential applications in food and medical industries because of the higher levels of flavonoid constituents and good bioactivities. Flavonoids are widely distributed in genus *Paeonia*, especially in the flowers of the plants [15,16]. Therefore, in addition to the ornamental value, the flower of the genus *Paeonia* can also be used as the source of flavonoids, and used in the food and drug fields.

## Figures and Tables

**Table 1:** Weight and flavonoid contents of different ethanol concentration elution fractions.

Ethanol Concentration (%)	Weight (g)	Flavonoids Content (mg·g^−1^)
20	0.83	43.4 ± 5.21
30	1.25	81.4 ± 3.26
40	2.21	108.5 ± 7.14
50	10.24	276.0 ± 6.58
60	7.62	257.8 ± 10.23
70	4.22	124.5 ± 8.56
80	2.67	61.4 ± 3.12
90	0.48	47.8 ± 2.89

**Table 2:** Scavenging effects of flavonoids from *P. ostii* on radicals.

Compounds	IC_50_ (μg·mL^−1^)			
	ABTS	O•^2−^	DPPH•	•OH
**1**	25.4 ± 0.68	75.8 ± 0.11	24.6 ± 0.41	141.9 ± 0.17
**2**	36.5 ± 0.76	86.4 ± 0.21	34.2 ± 0.98	155.2 ± 0.25
**3**	41.1 ± 0.24	84.2 ± 0.09	40.1 ± 0.13	157.3 ± 0.17
**4**	35.7 ± 0.35	80.1 ± 0.14	35.3 ± 0.21	134.8 ± 0.12
**5**	23.5 ± 0.44	69.4 ± 0.17	20.9 ± 0.27	121.8 ± 0.14
**Vc**	28.7 ± 2.54	72.3 ± 2.56	26.4 ± 0.21	132.1 ± 3.15

**Table 3:** Results of the determination of samples (mg·g^−1^).

Samples	1	2	3	4	5
Flavonoid-rich extracts	32.18 ± 1.21	84.75 ± 1.24	183.42 ± 2.74	106.25 ± 3.15	66.17 ± 2.23
Dried flower	0.62 ± 0.08	1.66 ± 0.34	10.25 ± 0.35	6.44 ± 0.45	1.02 ± 0.08
